# Transgenic *Metarhizium pingshaense* synergistically ameliorates pyrethroid-resistance in wild-caught, malaria-vector mosquitoes

**DOI:** 10.1371/journal.pone.0203529

**Published:** 2018-09-07

**Authors:** Etienne Bilgo, Brian Lovett, Koama Bayili, Abel Souro Millogo, Issiaka Saré, Roch K. Dabiré, Antoine Sanon, Raymond J. St. Leger, Abdoulaye Diabate

**Affiliations:** 1 Institut de Recherche en Sciences de la Santé, Centre Muraz, Bobo-Dioulasso, Burkina Faso; 2 Laboratoire d’Entomologie Fondamentale et Appliqué, UFR-SVT, Université Ouaga I, Pr. Joseph KI-Zerbo, Ouagadougou, Burkina Faso; 3 Department of Entomology, University of Maryland, College Park, Maryland, United States of America; 4 Université Nazi Boni, Bobo Dioulasso, Burkina Faso; Universita degli Studi di Camerino, ITALY

## Abstract

Transgenic *Metarhizium pingshaense* expressing the spider neurotoxin Hybrid (Met-Hybrid) kill mosquitoes faster and at lower spore doses than wild-type strains. In this study, we demonstrate that this approach dovetails with the cornerstone of current malaria control: pyrethroid-insecticides, which are the cornerstone of current malaria control. We used World Health Organization (WHO) tubes, to compare the impact on insecticide resistance of Met-Hybrid with red fluorescent *M*. *pingshaense* (Met-RFP), used as a proxy for the wild-type fungus. Insecticides killed less than 20% of *Anopheles coluzzii* and *Anopheles gambiae s*.*s*. mosquitoes collected in a malaria endemic region of Burkina Faso where pyrethroid use is common. Seven days post-infection, mortality for insecticide-sensitive and resistant mosquitoes averaged 94% with Met-Hybrid and 64% with Met-RFP, with LT_80_ values of 5.32±0.199 days and 7.76±0.183 days, respectively. Eighty nine percent of insecticide-resistant mosquitoes exposed to permethrin five days post-infection with Met-Hybrid died within 24 hours: only 22% died from Met-Hybrid alone over this 24-hour period. Compared to Met-RFP, Met-Hybrid also significantly reduced flight capacity of mosquitoes 3 to 5 days post-infection. Based on WHOPES phase I laboratory susceptibility bioassays, transgenic Met-Hybrid provides effective biological control for adult African malaria vectors that may be used to synergistically manage insecticide resistance with current methods.

## Background

An estimated 2 billion people live in areas where mosquito borne diseases are endemic. In 2015, malaria infected an estimated 212 million people around the world, killing 429,000, primarily children and pregnant women in sub-Saharan Africa [1]. Pyrethroid insecticide treated bed nets (ITNs) and indoor residual spraying (IRS) are current mainstays of mosquito control programs and have facilitated impressive reductions in the global malaria burden [2]. However, these approaches are threatened by increasing resistance to insecticides [3]. The widespread emergence of ‘knockdown resistance’ (kdr) in African mosquitoes was associated with the early intensive use of DDT and pyrethroids in agriculture, as both insecticides share the same mode of action [3–6]. In May 2012, the World Health Organization (WHO) launched the Global Plan for Insecticide Resistance Management in Malaria Vectors (GPIRM) with five strategic pillars(7). The third pillar highlighted a medium-term (3 to 10 year) need for an improved understanding of insecticide resistance, development of tools to manage resistance, as well as strategies for deploying these tools to achieve sustainable vector control. To meet these objectives, we are developing multiple alternative tools and strategies for exploiting transgenic mosquitoes and transgenic *Metarhizium* [7–9].

Entomopathogenic fungi are being evaluated as an environmentally friendly alternative to chemical insecticides [10]. However, compared to chemical insecticides, entomopathogenic fungi are considered to have poor efficacy (slow kill, high dose requirement, poor persistence in the field due to abiotic stressors) [11]. The large spore dose required also increases the difficulty of achieving sufficient coverage of mosquito resting surfaces or sleeping spaces so as to target enough adult vectors for effective reductions in disease burden.

To remedy these constraints, *Metarhizium* spp. targeting different insects have been engineered with improved tolerance to environmental stress [12]. Moreover, a strain of *Metarhizium* has been engineered to block transmission of malaria parasites *in insect* by expressing a single-chain antibody fragment targeting the parasite [13]. However, most genetic engineering efforts have aimed to increase fungal virulence. A transgenic, broad host-range *M*. *anisopliae* strain expressing an insect neurotoxin (AaIT) from *Androctonus australis* reduced mobility and blood feeding interest of adult *Aedes aegypti* mosquitoes, with a 9-fold reduction in lethal spore dose [14]. Similarly, a strain of *M*. *pingshaense* expressing the spider toxin Hybrid was highly effective against wild-caught, insecticide-resistant *Anopheles gambiae* s.l., the main vector of malaria transmission in Africa [9]. We chose Hybrid, also known as Versitude^TM^, for further development as it is effective and a well-characterized toxin: the US EPA has already approved it for use as a stand-alone insect control agent [15]. Furthermore, while pyrethroids and other chemical insecticides target neuronal voltage-gated sodium (Na_V_) channels. The Ca_V_ and K_V_ channels targeted by Hybrid are previously unexploited insecticide targets, reducing the likelihood of pre-existing resistance.

We envision genetically modified (GM) *Metarhizium* being used in conjunction with other technologies, including ITNs that are still the gold standard of vector control tools, even though pyrethroid resistance is now at high enough levels to render ITNs largely ineffective. Ten hours of exposure to permethrin produced a mortality rate in Burkina Faso *An*. *gambiae* of 26% as compared to an LT_50_ <2 min for the susceptible laboratory strain [16]. In laboratory studies, Met-Hybrid is equally infectious to insecticide-resistant and susceptible mosquitoes(9). Current proposals for field release involve application of the fungus to black cotton sheets which can be hung in houses to provide a resting site for mosquitoes that have taken blood meals [17]. This is the same application site as chemical insecticides, which are applied to house walls or used to treat the ITNs, so mosquitoes will likely be simultaneously confronted by both the fungus and pyrethroids. To successfully combine transgenic biopesticides and chemical insecticides in an integrated pest management program, it is necessary to determine if concurrent exposure synergizes their activities and better manages insecticide resistance in malaria vectors.

In this study, we adapted standard WHO laboratory bioassays for insecticides to compare the efficacy of the parental wild type strain and transgenic Met-Hybrid against wild mosquitoes with and without commonly used insecticides [18]. Our results suggest that transgenic fungi and insecticides would complement each other in field application and significantly mitigate the buildup of resistance when co-applied.

## Methods

### Ethical considerations

Ethical permissions required for this study were obtained through the Institutional Review of Institut de Recherche en Science de la Santé (IRSS) and Centre Muraz ethics committee (A012-2014/CE-CM).

### Mosquito colonies

For bioassays, we used F1 progeny of *An*. *coluzzii* and *An*. *gambiae s*.*s*. reared from larval collections at Kou Valley (11°23’ N, 4°24’ W) and Soumousso (11°04’ N, 4°03’ W), respectively. Mosquitoes from these areas are highly resistant to multiple insecticides [19]. We used the laboratory Kisumu colony of *Anopheles gambiae s*.*s*. as a pyrethroid-susceptible reference strain. Only non-blood-fed females, 2 to 5 days old, were used in bioassays. Approximately 100 mosquitoes (4 replicates of 25 mosquitoes each) were exposed to each fungal strain or a control without spores (Bioassays 1 and 2). All tests were carried out at 25°C±2°C and 80%±10% relative humidity.

### PCR determination of *kdr* levels

The level of *kdr* resistance within a subsample of mosquitoes was performed using the PCR protocol and primer sequences previously described [20]. We only analyzed mutation L1014F because it is the commonest in West Africa, whereas the L1014S mutation is confined to East Africa [21]. The primers AgD1 (5′-ATA GAT TCC CCG ACC ATG-3′) and AgD3 (5′-AAT TTG CAT TAC TTA CGA CA-3′) amplified the resistant allele yielding 195 bp fragments. The susceptible allele was assayed using primers AgD2 (5′-AGA CAA GGA TGA TGA ACC-3′) and AgD4 (5′-CTG TAG TGA TAG GAA ATT TA-3′), which amplified a 137 bp fragment. The primer set AgD1 and AgD2 amplified a ubiquitous 293 bp fragment as a positive control. During amplification, denaturation was set at 94°C for 3 min followed by 35 cycles of denaturation, annealing and elongation (94°C for 30 s, 55°C for 30 s, 72°C for 10 s, respectively). The final elongation was set at 72°C for 5 min.

### Fungal strains

An East African isolate of *Metarhizium pingshaense* was transformed to express either red fluorescent protein (RFP) or green fluorescent protein (GFP) under a constitutive promoter (GdpA). The GFP-expressing strains was also transformed to express Hybrid-toxin under the hemolymph-specific Mcl1 promoter [9]. The RFP-expressing strain demonstrates wild-type virulence and growth, and was used as a proxy for the wild type. Both strains were maintained on potato dextrose agar at 26°C with 70% relative humidity.

### Fungal formulations for bioassays

Conidia were formulated in organic sesame oil for application onto black cotton cloth; both the oil and the cloth were obtained in the local market in Bobo-Dioulasso, Burkina Faso. Five mL of a 8.0x10^7^ conidia/mL spore suspension containing 8% (v/v) sesame oil was spread onto 12 cm x 15 cm sections of black cloth to achieve ~2 × 10^6^ conidia/cm^2^. Impregnated sheets were dried for 16 hours overnight at ambient temperature (26±1°C, 80±10% RH) before placement inside WHO bioassay tubes (15 cm long and 4 cm diameter).

### First bioassay: Comparing the efficacy of fungi and pyrethroids to mosquitoes in WHO bioassay tubes

The insecticide-susceptible reference strain (*An*. *gambiae s*.*s*. Kisumu) and mosquitoes reared from larvae collected at field sites were bioassayed in WHO tubes following standard WHO procedures [18]. The tubes were loaded either with cotton cloths impregnated with Met-Hybrid or Met-RFP conidia (to infect mosquitoes with fungus), or test papers (supplied by the Universiti Sains Malaysia, Penang) impregnated with recommended dosages of permethrin (0.75%) or deltamethrin (0.05%). Control batches of mosquitoes were exposed to blank test papers (without insecticide) and/or to cotton sheets treated with an oil preparation without fungal spores. For each treatment and mosquito strain, 25 mosquitoes were used per replicate. This first bioassay was performed over 4 replicates, ~120 mosquitoes / treatment /mosquito strain.

After one hour of exposure to treated or untreated surfaces, mosquitoes were transferred to holding tubes and fed with 6% glucose. Fungal infection alone does not cause the instantaneous knockdown and mortality seen in insecticide-susceptible mosquitoes treated with chemical insecticides [20]. Beyond this, we recorded mortality daily over one week. For all bioassays, we observed fluorescent mycosis on cadavers to confirm death due to fungal treatments. Post-mortem, the legs of 3–5 days post-infection mosquitoes were removed and used for the Kdr frequency determination.

### Second bioassay: The impact of fungal infection on the susceptibility of mosquitos to permethrin

Mosquitoes were exposed to fungal spores (Met-RFP and Met-Hybrid) at ~2x10^6^ conidia/cm^2^ using the adapted WHO 2013 protocol described previously(9). Briefly, black cotton cloth impregnated with an oil formulation of conidia was stapled to cardstock for support and placed into a WHO tube with the cardstock facing outward. Mosquitoes were placed in the WHO tube for 1 hour to allow reliable fungal infection. One to five days after fungal infection, mosquitoes were placed in WHO tubes containing test paper impregnated with 0.75% permethrin. For each day post infection, an overall 120-mosquitoes/treatment/mosquito strain were used over 4 replicates for this second bioassay. Permethrin was chosen for this second bioassay because it is the main insecticide used on insecticide-treated bed nets (ITNs) currently mass distributed in Burkina Faso [2]. Any mosquito that died, became immobile and/or lost any part of any appendages before being transferred to the insecticide treatment was discarded according to WHOPES, 2013 requirements [18]. We followed knock-down during the course of a one-hour exposure time, after which mosquitoes were transferred to cages and mortality was followed for a further 24 hours. Mortalities were compared with both control batches of infected mosquitoes exposed to blank untreated paper and control batches of uninfected mosquitoes directly exposed to permethrin or to control paper. Wild-caught *An*. *gambiae s*.*s*., wild-caught *An*. *coluzzii* and lab-reared *An gambiae Kisumu* were used for this experiment.

### Third bioassay: Irritability cone tests on the impact of fungal infection on the flight capacity of mosquitoes

Insecticides, such as pyrethroids and DDT, have irritant and excito-repellent properties beyond their knock-down effects. Knockdown and irritant effects on *An coluzzii* resulting from tarsal contact with netting were measured one through five days post-infection with Met-RFP or Met-Hybrid. The fibers of treated netting (Olyset Plus^®^, Sumitomo Chemicals Co. Ltd., Japan) incorporate 2% (w/w) permethrin combined with 1% piperonyl butoxide (PBO). PBO works synergistically with permethrin in order to counteract metabolic-based pyrethroid resistance of mosquitoes [22]. For each replicate, six WHO plastic cones were attached to each 25 cm × 25 cm piece of treated or untreated (PBO alone) netting and held together by two plastic boards, which were clamped together with two binder clips. The assembly was held flat on the table (S1 Fig). Using an aspirator, one non-blood-fed female aged two to five days was gently introduced into each cone and the entrances were plugged with pieces of cotton. Each mosquito was exposed for 5 min. During the exposure period, two observers used electronic timers (one for landing and one for flying) to record the amount of time each mosquito spent flying and resting on the netting. After the 5 min test period each mosquito was transferred into a labeled 150 ml plastic holding cup and provided with 6% sugar solution. Knockdown and mortality were recorded 60 min and 24 h after exposure.

### Data management and analysis

All data were entered into Microsoft Windows Excel 2010, checked for accuracy, then imported to R studio version 2.11.1 for data manipulation, visualization and statistical analysis. (**S1 File**). Using Fisher’s exact test, P<0.05 was accepted as statistically significant. The susceptibility of the mosquitoes was evaluated on the basis of the 2013 WHOPES criteria of test mortality. Mosquitoes were considered to be alive if they could stand upright. A mosquito was classified as moribund if it cannot stand, cannot fly in a coordinated manner or takes off briefly, but falls immediately. A mosquito was classified as dead if were immobile, cannot stand or otherwise shows no signs of life [23]. The datasets generated during the current study, R codes (scripts) for all statistical analysis and visualizations (graphs) are available in this GitHub repository at https://github.com/EtienneBilgo/Transgenic_Metarhizium_Insecticides.

## Results

### Mosquito susceptibility to fungi and pyrethroids in WHO bioassay tubes

**Fig 1:**
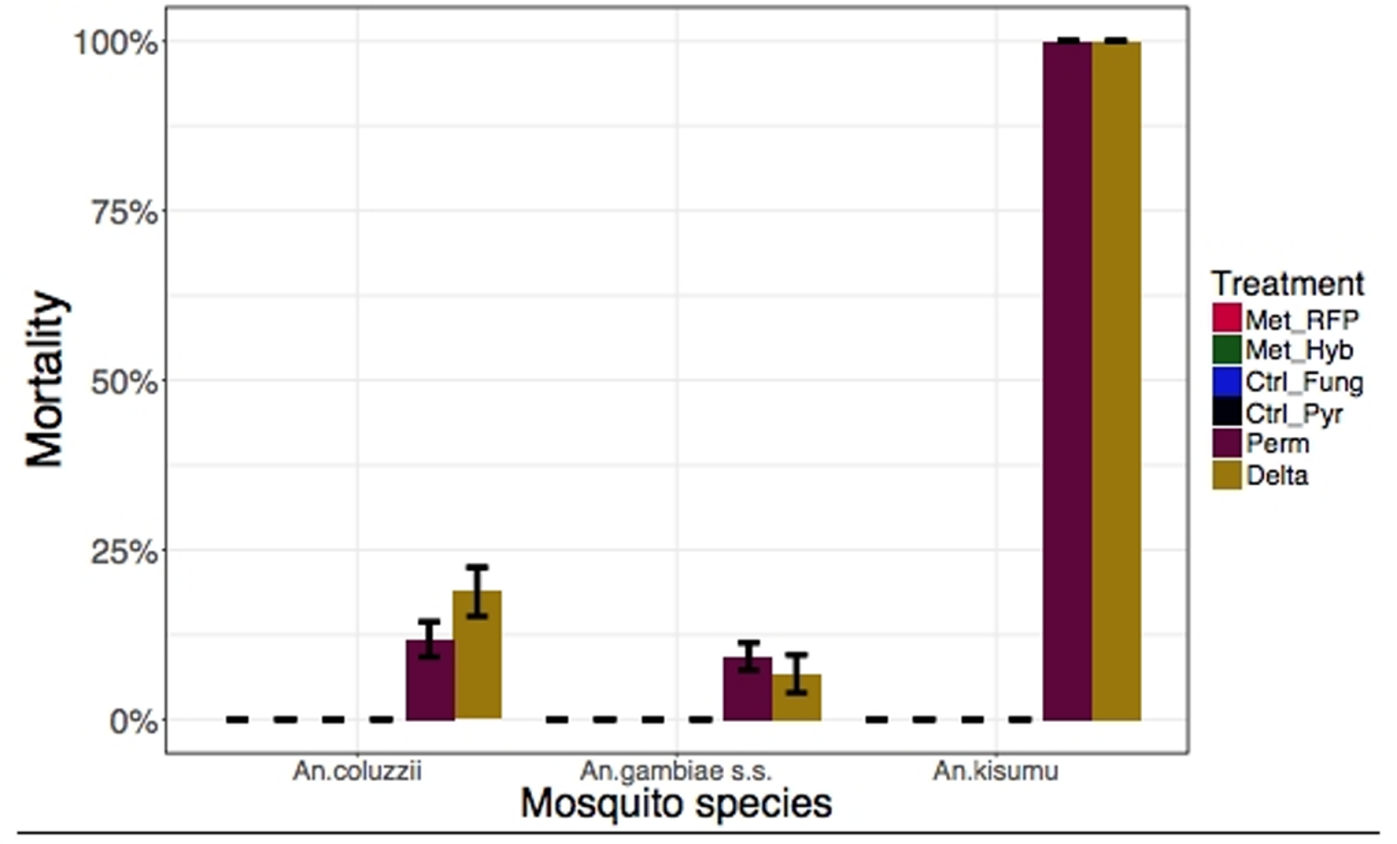
Results of World Health Organization (WHO) susceptibility tests for wild caught mosquitoes from Burkina Faso and laboratory *Anopheles gambiae Kisumu strain*. Adult female mosquitos were exposed to the WHO diagnostic dose of insecticides and *Metarhizium pingshaense* for 1 h, and mortality rates were recorded 24 h later.

**Fig 2:**
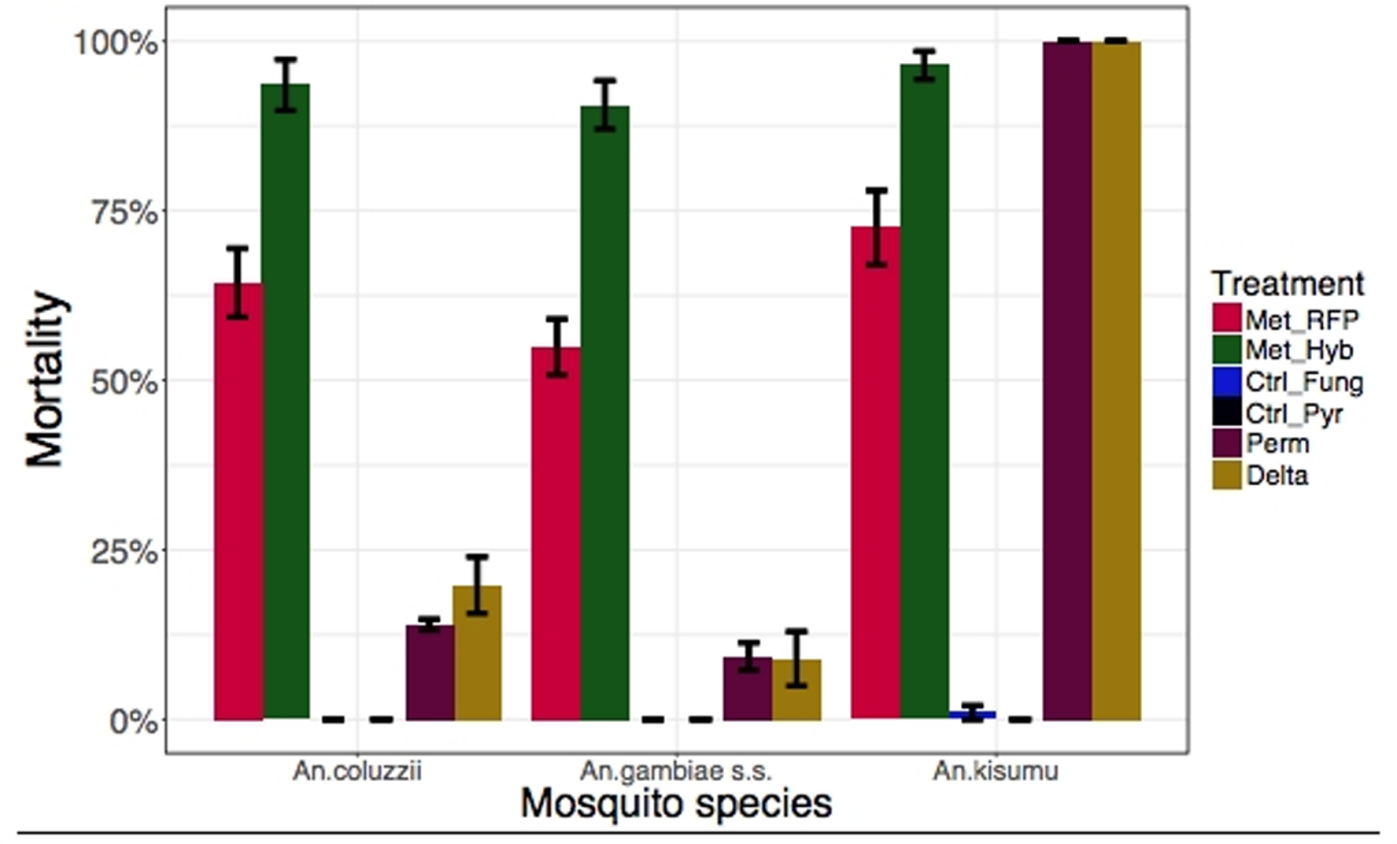
Results of World Health Organization (WHO) susceptibility tests for wild caught mosquitoes from Burkina Faso and laboratory *Anopheles gambiae Kisumu strain*. Adult female mosquitos were exposed to the WHO diagnostic dose of insecticides and *Metarhizium pingshaense* for 1 h, and mortality rates were recorded 7 days later.

As expected, Permethrin and Deltamethrin killed 100% of the insecticide-susceptible strain (*An*. *gambiae* Kisumu), confirming its status as a reference. We found that 96.9% of the *An*. *coluzzii* and 81.5% of the *An*.*gambiae s*.*s*. used for bioassays carried the *kdr* resistance gene (Table 1). Consequentially, they were far less susceptible to permethrin with 24-hour mortality of *An*. *coluzzii* and *An gambiae* being 11.8±2.58% and 9.30±2.03%, respectively. Likewise, Deltamethrin caused low mortality in both *An*. *coluzzii* (18.8±3.61%) and *An*. *gambiae s*.*s*. (6.72±2.81%) (Fig 1 and S1 Table). The surviving mosquitoes were monitored for a week during which time there was no significant difference between their mortality and controls that were not exposed to insecticides (Fig 2 and S1 Table).

**Table 1:** Kdr 1014L mutation frequency in tested wild-caught mosquitoes.

		Genotypes			
Species	N	1014L	1014L	1014F	*f (1014F (%))*
		1014L	1014F	1014F	
*An*. *coluzzii*	291	4	10	277	**0.969**
*An*.*gambiae s*.*s*.	279	35	33	211	**0.815**

Since *M*. *pingshaense* spores take 2–3 days to penetrate host cuticle, Met-RFP and Met-Hybrid do not cause mortality 24 hours post-infection (Fig 1 and S1 Table). However, within one week post-infection Met-Hybrid (Met-RFP) killed 93.5±3.77% (64.4±5.06%) of the *An*. *coluzzii*, 90.6±3.55% (54.9±4.10%) of the *An*.*gambiae s*.*s*. and 96.4±2.06% (72.5±5.50%) of the *An gambiae Kisumu*. As previously shown^9^, insecticide-resistance did not alter susceptibility to *Metarhizium* for both *An*. *coluzzii* and *An*. *gambiae* s.s. There were no statistical differences between the very high mortalities produced by Met-Hybrid and Permethrin or Deltamethrin in the insecticide sensitive *An gambiae Kisumu* mosquitoes (P = 0.31). Met-RFP was significantly less virulent than Met-Hybrid but significantly more effective than insecticides against insecticide-resistant mosquitoes (Fig 2 and S1 Table).

Met-Hybrid is the only treatment that achieved >80% mortality within one week (Table 2) with LT_80_’s of 5.18±0.482 days, 5.54±0.326 days and 5.25±0.269 days for *An*. *coluzzii*, *An*. *gambiae* and *An*. *Kisumu*, respectively. A mortality of 80% is the WHO threshold for successful vector control agents [23].

**Table 2:** LT_80_ survival following WHO tube exposure for various treatments according to mosquito species. SE: Standard error of the mean; Pairwise comparison of LT_80_ Treatments without letters in common are significant at P<0.05.

Mosquito species	Treatment	LT_80_ Mean (Days)	SE	Grouping
***Anopheles coluzzii***	Met_RFP	7.63	0.295	a
	Met_Hybrid	5.18	0.482	b
	Deltamethrin	-	-	-
	Permethrin	-	-	-
	Control1	-	-	-
	Control2	-	-	-
***Anopheles gambiae s*.*s*. (Giles)**	Met_RFP	8.12	0.358	a
	Met_Hybrid	5.54	0.326	b
	Deltamethrin	-	-	-
	Permethrin	-	-	-
	Control1	-	-	-
	Control2	-	-	-
***Anophles gambiae Kisumu***	Met_RFP	7.52	0.288	a
	Met_Hybrid	5.26	0.269	b
	Deltamethrin	0.769	0	c
	Permethrin	0.769	0	c
	Control1	-	-	-
	Control2	-	-	-

### Impact of fungal infection on the susceptibility of mosquitos to permethrin

In a previous study, we have reported that within 2.5 days post-infection, mosquitoes exposed to Met-Hybrid died faster than those exposed to Met-RFP with LT80 values for Met-Hybrid and Met-RFP were 4.14 ± 5.47 ± 0.25 and 7.71 ± 0.16 days, respectively (mean ± standard error is reported) [9]. The purpose of the current bioassay was to check the mortality rate of surviving fungal infected mosquitoes in contact with insecticide (Permethrin treated net) versus control-net (without any insecticide) following each day post infections during this range of time (1 to 5 days post-infection).

**Fig 3:**
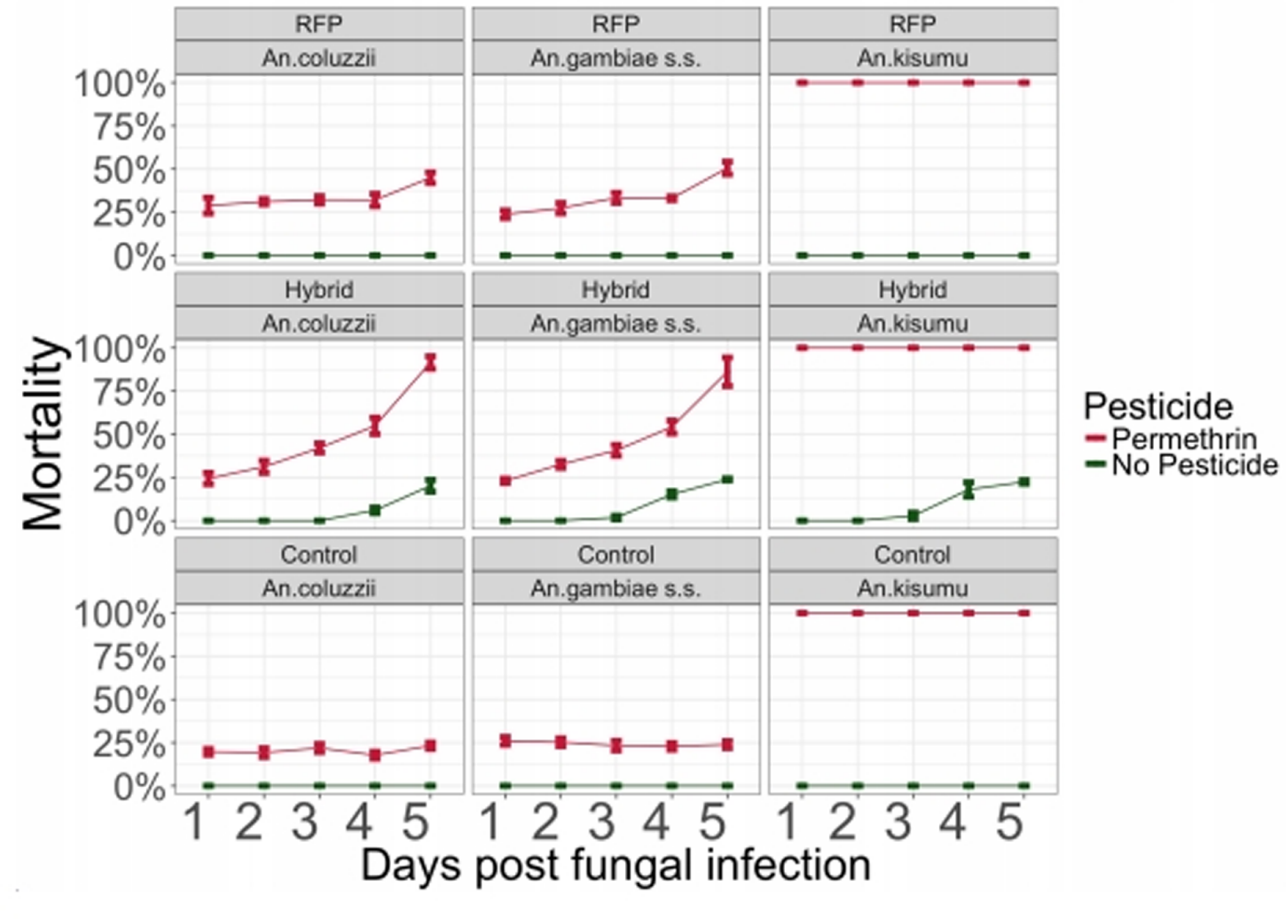
Legend. Curves of World Health Organization (WHO) susceptibility tests of the impact of fungal infections on the susceptibility of wild caught mosquitoes (*An*. *coluzzii* and *An*.*gambiae s*.*s*.) from Burkina Faso and laboratory *Anopheles gambiae Kisumu strain*. Following the first 5 days post-infection, living fungus-infected mosquitoes (Met_RFP and Met_Hybrid) versus uninfected mosquitoes (control) were exposed to the WHO diagnostic dose of permethrin treated net (pesticides) versus non-impregnated net (no pesticide) for 1 h, and mortality rates were recorded 24 h later.

We used WHO tubes and standard WHO protocols to test the effects of 0.75% permethrin on batches of mosquitoes one to five days post-infection. One hundred percent of the insecticide sensitive *An*. *gambiae kisumu* died at day 1 through 5 (Fig 3). The susceptibility of both *Anopheles coluzzii* and *Anopheles gambiae s*.*s*. to permethrin one-day post-infection with Met-Hybrid or Met-RFP was low (mean 28.3%) (S2 Table). Uninfected control mosquitoes retained a similar level of susceptibility (~22%) throughout the experiment (Fig 3). However, 88.8±4.28% of mosquitoes exposed to permethrin 5 days post-infection with Met-Hybrid died within 24 hours as compared to 23.5±1.26% that died from pesticides alone one day following pesticide exposure (a 3.78-fold increase) (Fig 3 and S2 Table). A 5-day infection with Met-RFP increased mortality of insecticide-susceptible mosquito 2.03-fold to 47.8±2.45%.

Between 3 to 5 days post-infection, significantly more (P<0.05) Met-Hybrid infected mosquitoes died within 24 hours of exposure to insecticide-free paper than those infected with Met-RFP (no mortality was observed in uninfected controls). Further, 3.9-fold more insecticide-resistant mosquitoes died (88.8±4.28% in total) 5 days-post-infection with Met-Hybrid and one day-post-exposure to permethrin than were killed by either Met-Hybrid (22.0±1.75%) or the permethrin (23.5±1.26%) used individually (Fig 3 and S2 Table). While the impact of Met-RFP infection with permethrin exceeds the sum of their impacts individually by day 5, this effect is observed earlier (starting day 2 post-infection) and to a greater extent mosquitoes are exposed to Met-Hybrid with permethrin. Fluorescent *Metarhizium* mycosis was observed on fungus-exposed cadavers, confirming mortality due to treatment.

### Effect of fungal infections on mosquitoes flying ability

**Fig 4:**
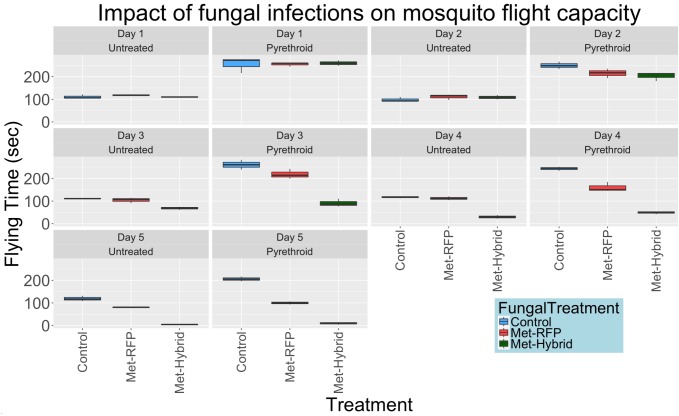
Results of World Health Organization (WHO) cone tests of the impact of fungal infections on the flight capacity of wild caught *Anopheles coluzzii* mosquitoes from Burkina Faso. Following the first 5 days post-infection, adult female mosquitos were exposed to untreated versus permethrin treated netting in the WHO cone for 5 minutes, and flying /landing times were recorded.

Fungal infection and Hybrid toxin expression may alter the ability of mosquitoes to sense and move away from chemical pesticides, thus altering exposure to pesticides on netting. Using WHO irritability cone tests, we individually assessed 15 mosquitoes per fungal treatment (Control, Met-RFP and Met-Hybrid) per day for 5 days against untreated and permethrin treated netting (a total of 450 mosquitoes). Uninfected mosquitoes took significantly (P<2.2x10^-6^) more flights when exposed to permethrin (S2 Table), confirming Permethrin irritates mosquitoes. Fig 4 represents how the first five days of fungal infection impacts flight capacity on both permethrin-treated and untreated netting. Overall, uninfected and infected mosquitoes spent less time flying with untreated netting as this lacked irritant and excito-repellent properties. Uninfected mosquitoes and mosquitoes infected for two days with Met-RFP or Met-Hybrid showed similar flying capacity (Table 3).

**Table 3:** Flight time on WHO irritability cone test on Permethrin treated versus untreated net following the first 5 days post-fungal infection. Following the first 5 days fungal post-infection, each female mosquito was exposed for 5 min on permethrin treated net versus untreated net. During the exposure period, the flying times were recorded.

Net Treatment	Days Post-Infection	Fungal Treatment	Percent of time in flight (%)	S E (%)
**Untreated**	Day 1	Met-Hybrid	36.7	0.52
		Control	37.0	1.71
		Met-RFP	39.5	0.64
	Day 2	Met-Hybrid	36.2	1.92
		Control	32.7	2.01
		Met-RFP	37.0	2.39
	Day 3	Met-Hybrid	22.8	1.36
		Control	37.0	0.15
		Met-RFP	34.8	2.34
	Day 4	Met-Hybrid	9.98	1.54
		Control	39.1	0.86
		Met-RFP	37.4	1.58
	Day 5	Met-Hybrid	1.49	0.41
		Control	39.7	2.24
		Met-RFP	26.6	0.45
**Permethrin**	Day 1	Met-Hybrid	86.5	2.33
		Control	85.0	6.58
		Met-RFP	84.8	1.72
	Day 2	Met-Hybrid	67.6	3.79
		Control	83.2	3.13
		Met-RFP	71.7	4.02
	Day 3	Met-Hybrid	30.3	3.36
		Control	86.9	4.20
		Met-RFP	72.8	4.23
	Day 4	Met-Hybrid	16.3	1.19
		Control	81.0	1.72
		Met-RFP	53.4	4.11
	Day 5	Met-Hybrid	3.27	1.13
		Control	68.6	1.99
		Met-RFP	33.3	1.35

Three to five days post-infection, when the fungus has entered the hemolymph and is expressing Met-Hybrid, mosquitoes infected with Met-Hybrid had significantly shorter flight times compared to uninfected controls and mosquitoes infected with Met-RFP (P<0.05; Table 3). Five days post-infection, mosquitoes infected with Met-RFP, but not exposed to pesticides, flew 13% less (~39 seconds) than the uninfected controls, and 25% (~75 seconds) more than mosquitoes infected with Met-Hybrid. These flight times differences were significantly different (P = 0.0045 for uninfected controls and P = 1.1x10^-5^ for Hybrid treated mosquitoes).

Fig 4 also illustrates the extent fungal infection impacts the flight capacity of mosquitoes exposed to permethrin treated netting. Met-Hybrid significantly reduced mosquito’s cumulative flight capacity 3 to 5 days post-infection compared to both uninfected controls and Met-RFP (Table 3). Five days-post-infection, mosquitoes infected with Met-Hybrid flew for 3.27±1.13% (9.80±3.40 seconds) of the bioassay, more than 10-fold less than mosquitoes infected by Met-RFP (33.3±1.35%, P = 8.7x10^-8^) or uninfected mosquitoes (68.6±1.99%, P = 8.9x10^-6^). The reduction in flight capacity due to Met-RFP (14.0% on Day 3 and 35.2% on Day 5) was significantly less (P < 0.05) than Met-Hybrid (56.6% on Day 3 and 65.3% on Day 5). By day 3 post-infection, there is no significant difference (P = 0.145) when comparing flight times between mosquitoes infected with Met-Hybrid on untreated (mean = 68.5) and permethrin treated net (mean = 90.8). However the flight times of mosquitoes infected with Met-RFP were significantly (P>0.05) higher on Permethrin treated net than untreated net each day following infection (Table 4). By day 3 post-infection, mosquitoes infected with Met-Hybrid no longer displayed irritability to Permethrin; however, these irritability effects were still observed in mosquitoes infected with Met-RFP and in control mosquitoes.

**Table 4:** Comparison of flight times of mosquitoes on untreated or permethrin treated net following each of the first 5 days post-fungal infection in a WHO cone bioassay.

	Day post- infection with fungi	Mean mosquito flight times (sec) untreated Net	Mean mosquito flight times (sec) Permethrin treated net	P value (Welch two samples t. test)
	1	110.9	255.0	0.01374
	2	98.13	249.5	0.0004312
**Control**	3	111.0	260.6	0.006944
	4	117.3	243.1	0.0002404
	5	119.1	205.7	0.0006898
	1	118.5	254.5	0.0004294
	2	110.9	215.0	0.003794
**Met-RFP**	3	104.3	218.5	0.003687
	4	112.2	160.1	0.04591
	5	79.87	100.0	0.02823
	1	110.1	259.5	0.001436
	2	108.6	202.9	0.005336
**Met-Hybrid**	3	68.53	90.80	0.1453
	4	29.93	49.00	0.03386
	5	4.467	9.800	0.2534

## Discussion

Current mosquito control technologies are increasingly inadequate, and novel technologies are needed to compliment them [10]. This will require a coordinated approach that integrates compatible technologies to build strengths synergistically and minimize risks. This is the first time that the efficacy of transgenic fungi against wild caught insecticide-resistant mosquitoes was compared with currently applied insecticides. The efficacy of the Hybrid-toxin against insecticide-resistant mosquitoes likely reflects Hybrid targeting two different neuron channels simultaneously in mosquitoes (Ca^2+^ and K^+^), compared to the single Na^+^ site targeted by permethrin and deltamethrin. S2 Fig. represents the mechanisms of action of Met-Hybrid and Pyrethroids. Mosquitoes exposed to WHO test papers impregnated with 0.4–1.6% insecticide pick up ~0.018–0.06μg insecticide/insect, respectively [24]. *M*. *pingshaense* spores are ~7μm by 3μm in size [25]. Estimating the volume of these spores using V = πr^2^h gives ~50 μm^3^. A yeast cell of this volume weighs ~53 pg [26,27]. The LD_100_ for Met-Hybrid is ~6 spores or ~318 pg of spores [9], which is ~58-fold less than the amount of insecticide picked up by a mosquito exposed to a 0.4–1.6% insecticide treated sheet.

Our results show that even with pyrethroid-resistant mosquitoes, transgenic fungi showed synergistic enhancement of insecticide-based control methods. It has already been shown that combining permethrin with wild-type fungi at very high concentrations increased mosquito mortality (*M*. *anisopliae* or *Beauveria bassiana* at 1×10^11^ spores/m^2^) [28]. Here we show that 5-days post-infection with a low spore dose (~130 transgenic *M*. *pingshaense* spores per mosquito) picked up from cloth impregnated with conidia increased the efficacy of permethrin or deltamethrin to insecticide resistant mosquitoes up to 91%. Previous studies on insect hosts other than mosquitoes have indicated fungi can act synergistically with insecticides [29]. Mixtures of *M*. *anisopliae* and deltamethrin displayed enhanced virulence when used together against ticks, indicating synergistic effects that would enhance effectiveness with lower concentrations of both deltamethrin and the fungus [29].

*Metarhizium* takes around 36 hours to breach the cuticle and enter the mosquito hemolymph [30]. The increased mortality of insecticide-resistant mosquitoes 3–5 days post inoculation with Met-Hybrid coincides with expression of the Hybrid-toxin in the mosquito hemolymph; the toxin is under the control of a hemolymph-specific promoter, so it is only expressed when the fungus is in contact with insect hemolymph [14]. Mosquitoes infected with Met-Hybrid showed accelerated loss of their flight capacity compared to those infected with Met-RFP. We previously used a blood feeding choice tunnel test to show that Hybrid-toxin significantly decreases the blood feeding propensity of mosquitoes within 3–5 days [9]. As the Hybrid-toxin acts on both sensory and motor neurons in the mosquito, reduced blood feeding is likely due to decreased sensation and mobility in Met-Hybrid infected mosquitoes.

Resistance to pyrethroids is typically mediated by the kdr genes. However, more potent biochemical, physiological and behavior resistance mechanisms have evolved and are now common in Burkina Faso [16,31]. In S1 Table, we report 11% and 18 of wild-caught *An*. *coluzzii* died 24 h post exposure to permethrin and Deltamethrin respectively. This suggests a small portion of *An*.*coluzzii* carrying the resistance gene (Kdr gene mutation, Table 1) demonstrated apparent susceptibility to insecticides in our bioassays: this may reflect limitations in the insecticide bioassays. Though following the WHO guidelines, the WHO cylinder Insecticidal bioassays are indeed sensitive to variations in temperature, humidity, time of day and physiological state, controlling for which necessitates standardized rearing and testing conditions [32]. Further, even though the Kdr mutation confers the resistance to pyrethroids, the correlation with WHO cone test mortality is not always identical and makes direct testing of wild-caught adult (female) mosquitoes problematic. Some studies suggested this gap could be readily filled by other molecular diagnostic markers, especially those targeting DNA [32] or new standardized bioassays that produce consistent dose-response measurements with a minimal number of mosquitoes [33]

In spite of this, Hybrid-expressing fungi significantly increased wild-caught mosquito susceptibly to insecticides: exceeding the 80% mortality threshold established by WHO [18]. The current study establishes that transgenic fungi meet the criteria established by GPIRM for malaria vector control, namely, they are suitable for combination in a mixture of IRS insecticides co-formulated into a single IRS product and can be combined with insecticide treated cloth or insecticide treated bed netting. Future modeling with transgenic fungi accounting for various mosquito ages and *Plasmodium* infection stages, as well as the coverage of ITN and IRS in communities, will facilitate the development of programs that combine transgenic fungi and chemical insecticides for malaria control in the field.

## Supporting information

S1 Fig The assembly of irritability cone tests to study the impact of fungal infection on the flight capacity of mosquitoes. (TIFF)

S1 File Supplemental R codes of analysis. (PDF)

S1 Table Mortality percentage of mosquitoes over 24 h and a week observation after 1 h exposure in WHO cylinder to insecticides and fungi. (DOCX)

S2 Table Results of World Health Organization (WHO) susceptibility tests of the impact of fungal infection on insecticide-susceptibility of wild caught mosquitoes from Burkina Faso and laboratory *Anopheles gambiae Kisumu strain*. (DOCX)

S2 Fig Schematic of hybrid activity on an insect neuron. (TIFF)
