# Supplementary figures and images for: Transgenic Metarhizium pingshaense synergistically ameliorates pyrethroid-resistance in wild-caught, malaria-vector mosquitoes
Source: PLoS One. 2018 Sep 7;13(9):e0203529. doi: 10.1371/journal.pone.0203529 (PMC6128571; doi:10.1371/journal.pone.0203529)

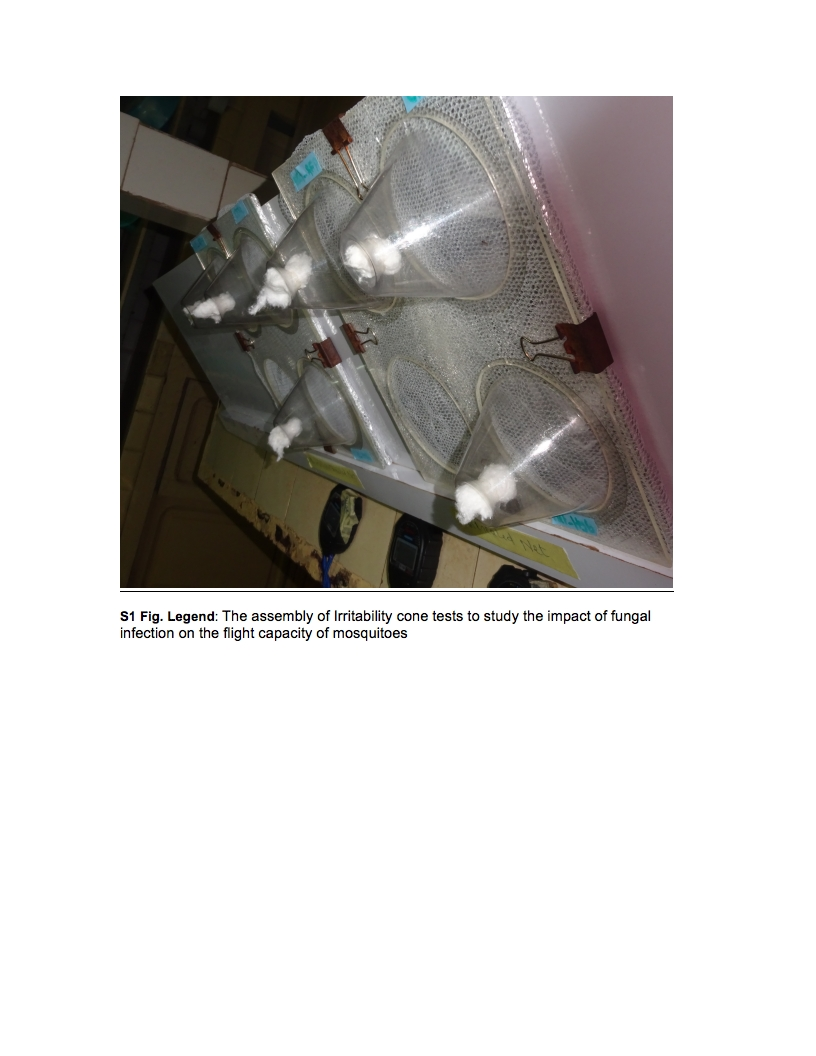

Supplement: S1 Fig — (TIFF) [file pone.0203529.s001.tiff]

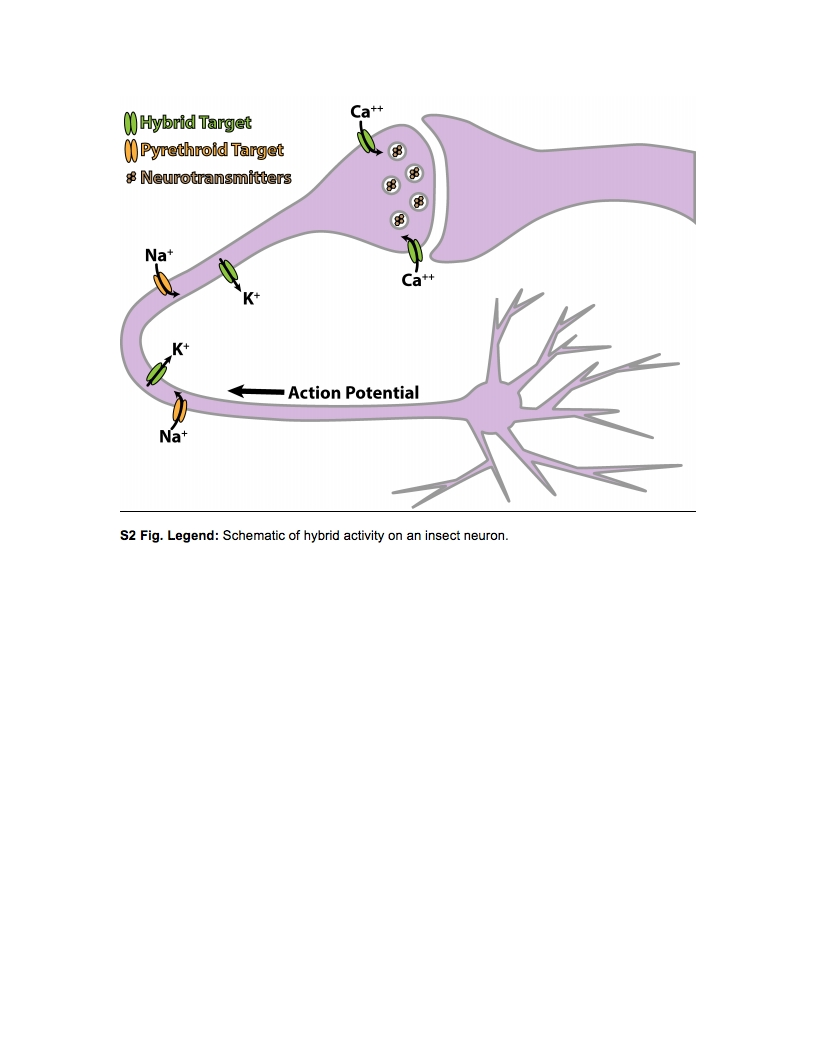

Supplement: S2 Fig — (TIFF) [file pone.0203529.s005.tiff]
