# Supplementary material for: Transgenic Metarhizium pingshaense synergistically ameliorates pyrethroid-resistance in wild-caught, malaria-vector mosquitoes
Source: PLoS One. 2018 Sep 7;13(9):e0203529. doi: 10.1371/journal.pone.0203529 (PMC6128571; doi:10.1371/journal.pone.0203529)
Supplement: S1 File — (PDF) [file pone.0203529.s002.pdf]

```r
#Transgenic Metarhizium pingshaense synergistically
# ameliorates pyrethroid-resistance in wild-caught, malaria-
#vector #mosquitoes
#14 March 2017
#Etienne Bilgo
#Centre Muraz/Institut de Recherche en Sciences de la Santé
#bilgo02@yahoo.fr
#Brian Lovett
#Department of Entomology
#University of Maryland
#lovettbr@umd.edu

#####Load packages####
library(MASS)
library(tidyverse)
library(reshape2)
library(plyr)
library(scales)
library(survival)
library(broom)

####Impact of Fungal Infection on Pesticide Susceptibility####
#Load data
S.dat <- read_csv("Infection_Susceptibility.csv")

#Data manipulation and tidying for analysis
S.dat$Day=as.factor(S.dat$Day)
S.dat$`Day post_inf`=as.factor(S.dat$`Day post_inf`)
S.dat$Species=as.factor(S.dat$Species)
S.dat=as.data.frame(S.dat)

# Melting data,reshape and make replicates colunm
S.dat2=melt(S.dat, colnames(S.dat)[c(1:2,27)],
 colnames(S.dat)[c(3:26)])
colnames(S.dat2)[c(2,4)]=c("DPI", "Fungus")

#Split combined variable
S.dat2$Replicate=S.dat2$Fungus
levels(S.dat2$Replicate)[grep("1",
 levels(S.dat2$Replicate))]="1"
levels(S.dat2$Replicate)[grep("2",
 levels(S.dat2$Replicate))]="2"
levels(S.dat2$Replicate)[grep("3",
 levels(S.dat2$Replicate))]="3"
levels(S.dat2$Replicate)[grep("4",
 levels(S.dat2$Replicate))]="4"
S.dat2$Pesticide=S.dat2$Fungus
```

```
levels(S.dat2$Pesticide)[grep("Ctrl",
 levels(S.dat2$Pesticide))]="No Pesticide"
levels(S.dat2$Pesticide)[grep("Hybrid",
 levels(S.dat2$Pesticide))]="Permethrin"
levels(S.dat2$Pesticide)[grep("RFP",
 levels(S.dat2$Pesticide))]="Permethrin"
levels(S.dat2$Pesticide)[grep("Perm",
 levels(S.dat2$Pesticide))]="Permethrin"
levels(S.dat2$Fungus)[grep("RFP",
 levels(S.dat2$Fungus))]="RFP"
levels(S.dat2$Fungus)[grep("Hyb",
 levels(S.dat2$Fungus))]="Hybrid"
levels(S.dat2$Fungus)[grep("Perm",
 levels(S.dat2$Fungus))]="Control"

#Analysis and visualization of of data of the impact on
 survival after contact with insecticides
S.dat3=ddply(S.dat2, .(Replicate, Fungus, Species, DPI,
 Pesticide), transform,
             Percent=cumsum(value)/sum(value), n=sum(value))
S.dat3=subset(S.dat3, Day!="Alive")
S.dat3$Day=as.numeric(as.character(S.dat3$Day))
S.dat4=ddply(S.dat3, .(Fungus, Species, DPI, Pesticide, Day),
 summarize, mean=mean(Percent),
             se=(sd(Percent)/sqrt(length(Percent))),
 Replicate=length(value))
levels(S.dat4$DPI)=1:5

S.dat5=S.dat4 %>%
  group_by(Fungus, Species, DPI, Pesticide, Day) %>%
  filter(Species!="An.kisumu" && Day==1) %>%
  group_by(Fungus, DPI, Pesticide) %>%
  do(summarise(., mean=mean(mean)))

S.Uninfected=mean(subset(S.dat5, Fungus=="Control" &
 Pesticide=="Permethrin")$mean)

S.dat6=S.dat3 %>%
  filter(Day==1 & Species!="An.kisumu") %>%
  group_by(Fungus, DPI, Pesticide) %>%
  do(summarize(.,mean=mean(Percent),
 se=(sd(Percent)/sqrt(length(Percent)))))

#Set aesthetics
theme = theme_bw()+theme(text = element_text(size=20),
 axis.title.x =element_text(size=30),
                         axis.text.x = element_text(hjust=1,
```

```
vjust=.5,size=35),
                        axis.text.y = element_text(size=25),
title =element_text(size=35),
                        legend.title = element_text(size=25),
legend.text= element_text(size=20),
                        legend.key = element_blank())
limits=aes(ymax=mean+se, ymin=mean-se)
cbPalette <-
c("#C70039","#155419","#0F18D0","#00010A","#5C063B","#9A770C")

#Visualize data
t.plt1=ggplot(subset(S.dat4, S.dat4$Day==1),aes(DPI, mean,
color=Pesticide))+

theme+scale_colour_manual(values=cbPalette)+geom_line(position
=position_dodge(0),aes(group=Pesticide))+
  geom_errorbar(limits, width=.2,
size=2,linetype="solid")+geom_point(position=position_dodge(0)
)+
  xlab("Days post fungal infection")+ylab(" Mortality
")+scale_y_continuous(labels=percent)+
  facet_wrap(~Fungus+Species)
t.plt1

t.plt2=ggplot(S.dat6, aes(DPI, mean, color=Pesticide))+

theme+scale_colour_manual(values=cbPalette)+geom_line(position
=position_dodge(0),aes(group=Pesticide))+
  geom_errorbar(limits, width=.2,
size=2,linetype="solid")+geom_point(position=position_dodge(0)
)+
  xlab("Days post fungal infection")+ylab(" Mortality
")+scale_y_continuous(labels=percent)+
  facet_wrap(~Fungus)
t.plt2

#Pull day 1 data only for statistics
D1.dat=subset(S.dat3, Day=="1")
D1.dat2=ddply(D1.dat, .(Fungus, Species, DPI),
summarize,mean=mean(Percent),

se=(sd(Percent)/sqrt(length(Percent))),Replicate=length(value)
)

#Pairwise t.tests

RFP.An.col.test=subset(D1.dat, Fungus=="RFP" &
```

```
 Species=="An.coluzzii" & Pesticide=="Permethrin")
pairwise.t.test(RFP.An.col.test$Percent, RFP.An.col.test$DPI,
 p.adj="none")

RFP.An.gam.test=subset(D1.dat, Fungus=="RFP" &
 Species=="An.gambiae s.s." & Pesticide=="Permethrin")
pairwise.t.test(RFP.An.gam.test$Percent, RFP.An.gam.test$DPI,
 p.adj="none")

RFP.An.kis.test=subset(D1.dat, Fungus=="RFP" &
 Species=="An.kisumu" & Pesticide=="Permethrin")
pairwise.t.test(RFP.An.kis.test$Percent, RFP.An.kis.test$DPI,
 p.adj="none")

Hybrid.An.col.test=subset(D1.dat, Fungus=="Hybrid" &
 Species=="An.coluzzii" & Pesticide=="Permethrin")
pairwise.t.test(Hybrid.An.col.test$Percent,
 Hybrid.An.col.test$DPI,  p.adj="none")

Hybrid.An.gam.test=subset(D1.dat, Fungus=="Hybrid" &
 Species=="An.gambiae s.s." & Pesticide=="Permethrin")
pairwise.t.test(Hybrid.An.gam.test$Percent,
 Hybrid.An.gam.test$DPI,  p.adj="none")

Hybrid.An.kis.test=subset(D1.dat, Fungus=="Hybrid" &
 Species=="An.kisumu" & Pesticide=="Permethrin")
pairwise.t.test(Hybrid.An.kis.test$Percent,
 Hybrid.An.kis.test$DPI,  p.adj="none")

Control.An.col.test=subset(D1.dat, Fungus=="Control" &
 Species=="An.coluzzii" & Pesticide=="Permethrin")
pairwise.t.test(Control.An.col.test$Percent,
 Control.An.col.test$DPI,  p.adj="none")

Control.An.gam.test=subset(D1.dat, Fungus=="Control" &
 Species=="An.gambiae s.s." & Pesticide=="Permethrin")
pairwise.t.test(Control.An.gam.test$Percent,
 Control.An.gam.test$DPI,  p.adj="none")

Control.An.kis.test=subset(D1.dat, Fungus=="Control" &
 Species=="An.kisumu" & Pesticide=="Permethrin")
pairwise.t.test(Control.An.kis.test$Percent,
 Control.An.kis.test$DPI,  p.adj="none")

C.RFP.An.col.test=subset(D1.dat, Fungus=="RFP" &
 Species=="An.coluzzii" & Pesticide=="No Pesticide")
pairwise.t.test(C.RFP.An.col.test$Percent,
```

```
 C.RFP.An.col.test$DPI,  p.adj="none")

C.RFP.An.gam.test=subset(D1.dat, Fungus=="RFP" &
 Species=="An.gambiae s.s." & Pesticide=="No Pesticide")
pairwise.t.test(C.RFP.An.gam.test$Percent,
 C.RFP.An.gam.test$DPI,  p.adj="none")

C.RFP.An.kis.test=subset(D1.dat, Fungus=="RFP" &
 Species=="An.kisumu" & Pesticide=="No Pesticide")
pairwise.t.test(C.RFP.An.kis.test$Percent,
 C.RFP.An.kis.test$DPI,  p.adj="none")

C.Hybrid.An.col.test=subset(D1.dat, Fungus=="Hybrid" &
 Species=="An.coluzzii" & Pesticide=="No Pesticide")
pairwise.t.test(C.Hybrid.An.col.test$Percent,
 C.Hybrid.An.col.test$DPI,  p.adj="none")

C.Hybrid.An.gam.test=subset(D1.dat, Fungus=="Hybrid" &
 Species=="An.gambiae s.s." & Pesticide=="No Pesticide")
pairwise.t.test(C.Hybrid.An.gam.test$Percent,
 C.Hybrid.An.gam.test$DPI,  p.adj="none")

C.Hybrid.An.kis.test=subset(D1.dat, Fungus=="Hybrid" &
 Species=="An.kisumu" & Pesticide=="No Pesticide")
pairwise.t.test(C.Hybrid.An.kis.test$Percent,
 C.Hybrid.An.kis.test$DPI,  p.adj="none")

C.Control.An.col.test=subset(D1.dat, Fungus=="Control" &
 Species=="An.coluzzii" & Pesticide=="No Pesticide")
pairwise.t.test(C.Control.An.col.test$Percent,
 C.Control.An.col.test$DPI,  p.adj="none")

C.Control.An.gam.test=subset(D1.dat, Fungus=="Control" &
 Species=="An.gambiae s.s." & Pesticide=="No Pesticide")
pairwise.t.test(C.Control.An.gam.test$Percent,
 C.Control.An.gam.test$DPI,  p.adj="none")

C.Control.An.kis.test=subset(D1.dat, Fungus=="Control" &
 Species=="An.kisumu" & Pesticide=="No Pesticide")
pairwise.t.test(C.Control.An.kis.test$Percent,
 C.Control.An.kis.test$DPI,  p.adj="none")

####Pesticide or Fungal Survival####
pyr.dat <- read.csv("Pyrethroid_Mortality.csv", sep=",")
colnames(pyr.dat)

#melt(Data set, column names to keep, column names to
```

```
 restructure)
pyr.dat=melt(pyr.dat, colnames(pyr.dat)[1:3],
 colnames(pyr.dat)[4:9])

pyr.dat2=ddply(pyr.dat, .(Replicate, Mosq.species,variable),
 transform, nval=sum(value, na.rm=TRUE),
 Mortality=cumsum(value)/sum(value, na.rm=TRUE))
pyr.dat2=subset(pyr.dat2, Day!="Alive")
pyr.dat2$Day=as.numeric(as.character(pyr.dat2$Day))
pyr.dat3=ddply(pyr.dat2, .(Day, Mosq.species,variable),
 summarize, mean=mean(Mortality), replicates=length(Mortality),
 se=sd(Mortality)/sqrt(length(Mortality)))
colnames(pyr.dat3)[3]="Treatment"

limits=aes(ymax=1-(mean+se), ymin=1-(mean-se))
theme = theme_bw()+theme(text =
 element_text(size=20),axis.title.x = element_text(size=30),
 axis.text.x = element_text(angle=90, hjust=1, vjust=.5,
 size=20), axis.text.y = element_text(size=25), title =
 element_text(size=35), legend.title = element_text(size=25),
 legend.text = element_text(size=20))
cbPalette <-
 c("#C70039","#155419","#0F18D0","#00010A","#5C063B","#9A770C")

P.plt1=ggplot(pyr.dat3, aes(Day, 1-mean,
 color=Treatment))+geom_line(size=2)+
  geom_errorbar(limits, width=.3, size=1)+theme+
  scale_colour_manual(values=cbPalette)+xlab("Days post
 treatment")+
  ylab("Mortality")+scale_y_continuous(labels=percent)+

 scale_x_continuous(breaks=0:max(pyr.dat3$Day))+facet_wrap(~Mos
 q.species)
P.plt1

#Analysis  of graphs for 24 hours,7 days and 14 days
#Day 1
pyr.dat3.D1=subset(pyr.dat3,Day=="1")

limits=aes(ymax=mean+se, ymin=mean-se)
colnames(pyr.dat3.D1)
cbPalette <-
 c("#C70039","#155419","#0F18D0","#00010A","#5C063B","#9A770C")
theme = theme_bw()+theme(text = element_text(size=20),
 axis.title.x = element_text(size=30), axis.text.x =
 element_text(angle=0, hjust=.5, vjust=0, size=20), axis.text.y
 = element_text(size=25), title = element_text(size=35),
```

```
 legend.title = element_text(size=25), legend.text =
 element_text(size=20))

P.plt2=ggplot(pyr.dat3.D1,
 aes(Mosq.species,mean,fill=Treatment))+
  geom_bar(stat="identity", position="dodge")+
  geom_errorbar(limits, width=.4, size=2,
 position=position_dodge(.9))+

 theme+scale_fill_manual(values=c("#C70039","#155419","#0F18D0"
 ,"#00010A","#5C063B","#9A770C"))+
  scale_y_continuous(labels=percent)+xlab("Mosquito
 species")+ylab("Mortality")
P.plt2

#Day 7
pyr.dat3.D7=subset(pyr.dat3,Day=="7")

P.plt3=ggplot(pyr.dat3.D7,
 aes(Mosq.species,mean,fill=Treatment))+
  geom_bar(stat="identity", position="dodge")+
  geom_errorbar(limits, width=.4, size=2,
 position=position_dodge(.9))+

 theme+scale_fill_manual(values=c("#C70039","#155419","#0F18D0"
 ,"#00010A","#5C063B","#9A770C"))+
  scale_y_continuous(labels=percent)+xlab("Mosquito
 species")+ylab("Mortality")
P.plt3

pyr.dat3.D7.mn= pyr.dat3.D7 %>%
  group_by(Treatment) %>%
  do(summarize(.,mean=mean(mean)))

#Statistic and pairwise comparaison
#Pairwise over 1 week
#An. coluzzii
An.col.testdat.D7=subset(pyr.dat2, Day=="7" &
 Mosq.species=="An.coluzzii")
pairwise.t.test(An.col.testdat.D7$Mortality,
 An.col.testdat.D7$variable,  p.adj="none")

#An. gambiae s.s.
An.gam.testdat.D7=subset(pyr.dat2, Day=="7" &
 Mosq.species=="An.gambiae s.s.")
pairwise.t.test(An.gam.testdat.D7$Mortality,
 An.gam.testdat.D7$variable,  p.adj="none")
```

```
#An. kisumu
An.kis.testdat.D7=subset(pyr.dat2, Day=="7" &
 Mosq.species=="An.kisumu")
pairwise.t.test(An.kis.testdat.D7$Mortality,
 An.kis.testdat.D7$variable,  p.adj="none")

#Pairwise over 1 day
#An. coluzzii
An.col.testdat.D1=subset(pyr.dat2, Day=="1" &
 Mosq.species=="An.coluzzii")
pairwise.t.test(An.col.testdat.D1$Mortality,
 An.col.testdat.D1$variable,  p.adj="none")

#An. gambiae s.s.
An.gam.testdat.D1=subset(pyr.dat2, Day=="1" &
 Mosq.species=="An.gambiae s.s.")
pairwise.t.test(An.gam.testdat.D1$Mortality,
 An.gam.testdat.D1$variable,  p.adj="none")

#An. kisumu
An.kis.testdat.D1=subset(pyr.dat2, Day=="1" &
 Mosq.species=="An.kisumu")
pairwise.t.test(An.kis.testdat.D1$Mortality,
 An.kis.testdat.D1$variable,  p.adj="none")

#Treatments accoring to Mosquito species Day 7
#RFP
RFP.testdat.D7=subset(pyr.dat2, Day=="7" &
 variable=="Met_RFP")
pairwise.t.test(RFP.testdat.D7$Mortality,
 RFP.testdat.D7$Mosq.species,  p.adj="none")

#Hybrid
Hybrid.testdat.D7=subset(pyr.dat2, Day=="7" &
 variable=="Met_Hyb")
pairwise.t.test(Hybrid.testdat.D7$Mortality,
 Hybrid.testdat.D7$Mosq.species,  p.adj="none")

#Fungal control
F.C.testdat.D7=subset(pyr.dat2, Day=="7" &
 variable=="Ctrl_Fung")
pairwise.t.test(F.C.testdat.D7$Mortality,
 F.C.testdat.D7$Mosq.species,  p.adj="none")

#Pesticide Control
P.C.testdat.D7=subset(pyr.dat2, Day=="7" &
```

```
 variable=="Ctrl_Pyr")
pairwise.t.test(P.C.testdat.D7$Mortality,
 P.C.testdat.D7$Mosq.species,  p.adj="none")

#Permethrin
Per.testdat.D7=subset(pyr.dat2, Day=="7" & variable=="Perm")
pairwise.t.test(Per.testdat.D7$Mortality,
 Per.testdat.D7$Mosq.species,  p.adj="none")

#Deltamethrin
Del.testdat.D7=subset(pyr.dat2, Day=="7" & variable=="Delta")
pairwise.t.test(Del.testdat.D7$Mortality,
 Del.testdat.D7$Mosq.species,  p.adj="none")

#Treatments accoring to Mosquito species Day 1
#RFP
RFP.testdat.D1=subset(pyr.dat2, Day=="1" &
 variable=="Met_RFP")
pairwise.t.test(RFP.testdat.D1$Mortality,
 RFP.testdat.D1$Mosq.species,  p.adj="none")

#Hybrid
Hybrid.testdat.D1=subset(pyr.dat2, Day=="1" &
 variable=="Met_Hyb")
pairwise.t.test(Hybrid.testdat.D1$Mortality,
 Hybrid.testdat.D1$Mosq.species,  p.adj="none")

#Fungal control
F.C.testdat.D1=subset(pyr.dat2, Day=="1" &
 variable=="Ctrl_Fung")
pairwise.t.test(F.C.testdat.D1$Mortality,
 F.C.testdat.D1$Mosq.species,  p.adj="none")

#Pesticide Control
P.C.testdat.D1=subset(pyr.dat2, Day=="1" &
 variable=="Ctrl_Pyr")
pairwise.t.test(P.C.testdat.D1$Mortality,
 P.C.testdat.D1$Mosq.species,  p.adj="none")

#Permethrin
Per.testdat.D1=subset(pyr.dat2, Day=="1" & variable=="Perm")
pairwise.t.test(Per.testdat.D1$Mortality,
 Per.testdat.D1$Mosq.species,  p.adj="none")

#Deltamethrin
Del.testdat.D1=subset(pyr.dat2, Day=="1" & variable=="Delta")
pairwise.t.test(Del.testdat.D1$Mortality,
```

```
 Del.testdat.D1$Mosq.species,  p.adj="none")

#Calculate LT80s
LTdat.pyr=pyr.dat2
colnames(LTdat.pyr)[4]=c("Treatment")
LTdat.pyr$Dead=LTdat.pyr$nval*LTdat.pyr$Mortality
LTdat.pyr$Alive=LTdat.pyr$nval-LTdat.pyr$Dead
attach(LTdat.pyr)
surv.per=0.20
LTdat.pyr2=ddply(LTdat.pyr, .(Mosq.species, Treatment,
 Replicate), summarize,

 LT=as.numeric(dose.p(glm(cbind(Alive,Dead)~Day,binomial),p=sur
 v.per)))
LTdat.pyr2[LTdat.pyr2$LT>14 | LTdat.pyr2$LT<0,]$LT=NA
LT80.Error=ddply(LTdat.pyr2, .(Mosq.species, Treatment),
 summarize, "LT80 Mean"=mean(LT,na.rm=T), se=sd(LT,
 na.rm=T)/sqrt(length(LT[!is.na(LT)])),
 Replicates=length(LT[!is.na(LT)]))

LT80.Error.2=ddply(LTdat.pyr2, .(Treatment), summarize, "LT80
 Mean"=mean(LT,na.rm=T), se=sd(LT,
 na.rm=T)/sqrt(length(LT[!is.na(LT)])),
 Replicates=length(LT[!is.na(LT)]))

#Comparisons by mosquito species
#An. coluzzii
LT.col.testdat=subset(LTdat.pyr2, Mosq.species=="An.coluzzii"
 & !is.na(LT))
pairwise.t.test(LT.col.testdat$LT, LT.col.testdat$Treatment,
 p.adj="none")

#An. gambiae
LT.gam.testdat=subset(LTdat.pyr2, Mosq.species=="An.gambiae
 s.s." & !is.na(LT))
pairwise.t.test(LT.gam.testdat$LT, LT.gam.testdat$Treatment,
 p.adj="none")

#An. kisumu
LT.kis.testdat=subset(LTdat.pyr2, Mosq.species=="An.kisumu" &
 !is.na(LT))
pairwise.t.test(LT.kis.testdat$LT, LT.kis.testdat$Treatment,
 p.adj="none")

#Comparisons by treatment
#RFP
LT.RFP.testdat=subset(LTdat.pyr2, Treatment=="Met_RFP" &
```

```
 !is.na(LT))
pairwise.t.test(LT.RFP.testdat$LT,
 LT.RFP.testdat$Mosq.species, p.adj="none")

#Hybrid
LT.Hybrid.testdat=subset(LTdat.pyr2, Treatment=="Met_Hyb" &
 !is.na(LT))
pairwise.t.test(LT.Hybrid.testdat$LT,
 LT.Hybrid.testdat$Mosq.species, p.adj="none")

#####Irritability####
Ir <- read.csv("Infection_Irritability.csv", sep=",")

#Data manipulation and cleaning
colnames(Ir)=c("Replicate", "Species", "DaysPostInfection",
 "MosquitoNumber", "NetTreatment", "FungalTreatment",
 "TestingTime", "LandingTime", "FlightTime", "Flights",
 "Landings")
Ir$Replicate=as.factor(Ir$Replicate)
levels(Ir$NetTreatment)=c("Untreated", "Untreated",
 "Pyrethroid")
levels(Ir$FungalTreatment)=c("Control", "Control", "Met-
 Hybrid", "Met-RFP")
Ir$FungalTreatment =
 factor(Ir$FungalTreatment,levels(Ir$FungalTreatment)[c(1,3,2)]
 )

#Irritability comparisons for each day in control mosquitoes
#1 DPI
Ir.control.D1=subset(Ir, Ir$FungalTreatment=="Control" &
 Ir$DaysPostInfection==1)
pairwise.t.test(Ir.control.D1$Flights,
 Ir.control.D1$NetTreatment, p.adj="none")

#2 DPI
Ir.control.D2=subset(Ir, Ir$FungalTreatment=="Control" &
 Ir$DaysPostInfection==2)
pairwise.t.test(Ir.control.D2$Flights,
 Ir.control.D2$NetTreatment, p.adj="none")

#3 DPI
Ir.control.D3=subset(Ir, Ir$FungalTreatment=="Control" &
 Ir$DaysPostInfection==3)
pairwise.t.test(Ir.control.D3$Flights,
 Ir.control.D3$NetTreatment, p.adj="none")

#4 DPI
```

```
Ir.control.D4=subset(Ir, Ir$FungalTreatment=="Control" &
 Ir$DaysPostInfection==4)
pairwise.t.test(Ir.control.D4$Flights,
 Ir.control.D4$NetTreatment, p.adj="none")

#5 DPI
Ir.control.D5=subset(Ir, Ir$FungalTreatment=="Control" &
 Ir$DaysPostInfection==5)
pairwise.t.test(Ir.control.D5$Flights,
 Ir.control.D5$NetTreatment, p.adj="none")

Ir1=ddply(Ir, .(Replicate, Species, DaysPostInfection,
 NetTreatment, FungalTreatment), summarize,
 LandingTime.mn=mean(LandingTime),
 FlightTime.mn=mean(FlightTime), Flights.mn=mean(Flights),
 Landings.mn=mean(Landings),
 PercentFlying=sum(FlightTime)/sum(TestingTime))
Ir2=ddply(Ir1, .(DaysPostInfection, NetTreatment,
 FungalTreatment), summarize, Percent=mean(PercentFlying,
 na.rm=T), se=sd(PercentFlying,
 na.rm=T)/sqrt(length(PercentFlying)),
 Replicate=length(PercentFlying))

Untreated_Ir2=subset(Ir2,NetTreatment=="Untreated")

limits=aes(ymax=Percent+se, ymin=Percent-se)
theme = theme_bw()+theme(text = element_text(size=25),
 axis.title.x = element_text(size=30),title
 =element_text(size=35))

levels(Untreated_Ir2$FungalTreatment)
Untreated_Plt=ggplot(Untreated_Ir2,aes(DaysPostInfection,Perce
 nt,color=FungalTreatment))+geom_line(size=3)+geom_errorbar(lim
 its, width=.2, size=2, linetype="solid")+theme+xlab("Days post
 fungal infection")+ylab("Percent flying
 ")+scale_color_manual(values=c("#006400", "#49acff",
 "#ff4444"))
Untreated_Plt

#We can try boxplots representation for flight_time
Untreated_Irb=subset(Ir1,NetTreatment=="Untreated")

theme = theme_grey()+theme(text = element_text(size=20),
 axis.title.x = element_text(size=30), axis.text.x =
 element_text(angle=90, hjust=1, vjust=.5, size=20),
 axis.text.y = element_text(size=25), title =
 element_text(size=35), plot.title = element_text(hjust = 0.5),
```

```
 legend.title = element_text(size=25), legend.text =
 element_text(size=20),legend.background =
 element_rect(fill="lightblue",size=0.5,
 linetype="solid"),legend.position=c(0.8, 0.2))

Untreated_Irb$DaysPostInfection<-
 factor(Untreated_Irb$DaysPostInfection)
levels(Untreated_Irb$DaysPostInfection)
levels(Untreated_Irb$DaysPostInfection)[1]="Day 1"
levels(Untreated_Irb$DaysPostInfection)[2]="Day 2"
levels(Untreated_Irb$DaysPostInfection)[3]="Day 3"
levels(Untreated_Irb$DaysPostInfection)[4]="Day 4"
levels(Untreated_Irb$DaysPostInfection)[5]="Day 5"
View(Untreated_Irb)
colnames(Untreated_Irb)
Untreated.box<-ggplot(Untreated_Irb,aes(FungalTreatment,
 FlightTime.mn,
 fill=FungalTreatment))+geom_boxplot()+theme+xlab("Treatment")+
 ylab("Flying Time (sec) ")+ggtitle("Untreated
 net")+facet_wrap(~DaysPostInfection)+scale_fill_manual(values=
 c("#49acff", "#ff4444", "#006400"))
Untreated.box

#For treated net
Perm_Ir2=subset(Ir2,NetTreatment=="Pyrethroid")

limits=aes(ymax=Percent+se, ymin=Percent-se)
theme = theme_bw()+theme(text = element_text(size=25),
 axis.title.x = element_text(size=30),title
 =element_text(size=45))

Perm_Plt=ggplot(Perm_Ir2,aes(DaysPostInfection,Percent,color=F
 ungalTreatment))+geom_line(size=3)+geom_errorbar(limits,
 width=.2, size=2, linetype="solid")+theme+xlab("Days post
 fungal infection")+ylab("Percent flying
 ")+scale_color_manual(values=c("#49acff", "#ff4444",
 "#006400"))
Perm_Plt

Both_Plt=ggplot(Ir2,aes(DaysPostInfection,Percent,color=Fungal
 Treatment))+geom_line(size=3)+geom_errorbar(limits, width=.2,
 size=2, linetype="solid")+theme+xlab("Days post fungal
 infection")+ylab("Percent flying
 ")+facet_wrap(~NetTreatment)+scale_color_manual(values=c("#49a
 cff", "#ff4444",
 "#006400"))+scale_y_continuous(labels=percent, limits=c(0,1))
Both_Plt
```

```
#We can visualize flighting time on Permethrin treated net
 using boxplots flight time
B_Perm_Ir=subset(Ir1,NetTreatment=="Pyrethroid")

B_Perm_Ir$DaysPostInfection<-
 factor(B_Perm_Ir$DaysPostInfection)
levels(B_Perm_Ir$DaysPostInfection)
levels(B_Perm_Ir$DaysPostInfection)[1]="Day 1"
levels(B_Perm_Ir$DaysPostInfection)[2]="Day 2"
levels(B_Perm_Ir$DaysPostInfection)[3]="Day 3"
levels(B_Perm_Ir$DaysPostInfection)[4]="Day 4"
levels(B_Perm_Ir$DaysPostInfection)[5]="Day 5"

limits=aes(ymax=Percent+se, ymin=Percent-se)
theme = theme_grey()+theme(text = element_text(size=20),
 axis.title.x = element_text(size=30), axis.text.x =
 element_text(angle=90, hjust=1, vjust=.5, size=20),
 axis.text.y = element_text(size=25), title =
 element_text(size=30), plot.title = element_text(hjust = 0.5),
 legend.title = element_text(size=25), legend.text =
 element_text(size=20), legend.background =
 element_rect(fill="lightblue", size=0.5, linetype="solid"),
 legend.position=c(0.8, 0.01))

Perm.box<-
 ggplot(B_Perm_Ir,aes(FungalTreatment,FlightTime.mn,fill=Fungal
 Treatment))+geom_boxplot()+theme+xlab("Treatment")+
 ylab("Flying Time (sec) ")+ggtitle("Permethrin treated
 net")+facet_wrap(~DaysPostInfection)+scale_fill_manual(values=
 c("#49acff", "#ff4444", "#006400"))
Perm.box

Both.box<-ggplot(rbind(B_Perm_Ir,
 Untreated_Irb),aes(FungalTreatment,FlightTime.mn,fill=FungalTr
 eatment))+geom_boxplot()+theme+xlab("Treatment")+ ylab("Flying
 Time (sec) ")+ggtitle("Impact of fungal infections on mosquito
 flight
 capacity")+facet_wrap(~DaysPostInfection+NetTreatment)+scale_f
 ill_manual(values=c("#49acff", "#ff4444", "#006400"))
Both.box

#Pairwise comparisons of t.test
#For untreated net
#For day 1 post infection
t.test(Ir$Flights~Ir$NetTreatment)
```

```
day1_Untreated_Irb=subset(Untreated_Irb,DaysPostInfection=="Da
y 1")
pairwise.t.test(day1_Untreated_Irb$FlightTime,
 day1_Untreated_Irb$FungalTreatment,  p.adj="none")

#For day 2 post infection
day2_Untreated_Irb=subset(Untreated_Irb,DaysPostInfection=="Da
y 2")
pairwise.t.test(day2_Untreated_Irb$PercentFlying,
 day2_Untreated_Irb$FungalTreatment,  p.adj="none")

###For day 3 post infection
day3_Untreated_Irb=subset(Untreated_Irb,DaysPostInfection=="Da
y 3")
pairwise.t.test(day3_Untreated_Irb$PercentFlying,
 day3_Untreated_Irb$FungalTreatment,  p.adj="none")

#For day 4 post infection
day4_Untreated_Irb=subset(Untreated_Irb,DaysPostInfection=="Da
y 4")
pairwise.t.test(day4_Untreated_Irb$PercentFlying,
 day4_Untreated_Irb$FungalTreatment,  p.adj="none")

#For day 5 post infection
day5_Untreated_Irb=subset(Untreated_Irb,DaysPostInfection=="Da
y 5")
pairwise.t.test(day5_Untreated_Irb$PercentFlying,
 day5_Untreated_Irb$FungalTreatment,  p.adj="none")

#For treated net
Perm_Ir=subset(Ir1, Ir1$NetTreatment=="Pyrethroid")

#For day 1 post infection
day1_Perm_Ir=subset(Perm_Ir, DaysPostInfection=="1")
pairwise.t.test(day1_Perm_Ir$PercentFlying,
 day1_Perm_Ir$FungalTreatment,  p.adj="none")

#For day 2 post infection
day2_Perm_Ir=subset(Perm_Ir,DaysPostInfection=="2")
pairwise.t.test(day2_Perm_Ir$PercentFlying,
 day2_Perm_Ir$FungalTreatment,  p.adj="none")

#For day 3 post infection
day3_Perm_Ir=subset(Perm_Ir,DaysPostInfection=="3")
pairwise.t.test(day3_Perm_Ir$PercentFlying,
 day3_Perm_Ir$FungalTreatment,  p.adj="none")
```

```
#For day 4 post infection
day4_Perm_Ir=subset(Perm_Ir,DaysPostInfection=="4")
pairwise.t.test(day4_Perm_Ir$PercentFlying,
 day4_Perm_Ir$FungalTreatment,  p.adj="none")

#For day 5 post infection
day5_Perm_Ir=subset(Perm_Ir,DaysPostInfection=="5")
pairwise.t.test(day5_Perm_Ir$PercentFlying,
 day5_Perm_Ir$FungalTreatment,  p.adj="none")

#Comparing flight time between treated net and untreated net

Ir.tests= Ir1 %>%
  group_by(FungalTreatment, DaysPostInfection) %>%
  do(tidy(t.test(FlightTime.mn~NetTreatment, data= .))) %>%
  ungroup() %>%
  dplyr::select("Fungal Treatments"=FungalTreatment, "Days
 Post-infection with fungi"=DaysPostInfection, "Mean mosquito
 flight time on untreated net (sec)"=estimate1, "Mean mosquito
 flight time on Permethrin treated net (sec)"=estimate2, "p
 value"=p.value, "Method"=method)

####Insecticide Resistance####
R.dat <- read_csv("Resistance_Levels.csv")
R.dat$Village=as.factor(R.dat$Village)
R.dat$Species=as.factor(R.dat$Species)
R.dat$Kdr_West=as.factor(R.dat$Kdr_West)
R.dat2=ddply(R.dat, .(Species), summarize, N=length(Kdr_West),
 `1014L/1014L`=length(subset(Kdr_West, Kdr_West=="SS")),
 `1014L/1014F`=length(subset(Kdr_West, Kdr_West=="RS")),
 `1014F/1014F`=length(subset(Kdr_West, Kdr_West=="RR")))
R.dat2$`Frequency
1014F`=(2*R.dat2$`1014F/1014F`+R.dat2$`1014L/1014F`)/(2*R.dat2
$N)
```
