# Supplementary material for: Transgenic Metarhizium pingshaense synergistically ameliorates pyrethroid-resistance in wild-caught, malaria-vector mosquitoes
Source: PLoS One. 2018 Sep 7;13(9):e0203529. doi: 10.1371/journal.pone.0203529 (PMC6128571; doi:10.1371/journal.pone.0203529)
Supplement: S1 Table — (DOCX) [file pone.0203529.s003.docx]

| **Mosquito species** | **Treatments** | **% Mortality (24 hours)** | **Grouping (% Mortality 24 hours)** | **% Mortaliy**  **(1 week)** | **Grouping (% mortality 1 week)** |
| --- | --- | --- | --- | --- | --- |
| **An.**  **coluzzii** | Ctrl_Fung | 0 | a | 0 | a |
|  | Ctrl_Pyr | 0 | a | 0 | a |
|  | Met_RFP | 0 | a | 64.4±5.06 | b |
|  | Met_Hyb | 0 | a | 93.5±3.77 | c |
|  | Permethrin | 11.78 ± 2.58 | b | 14.0±0.777 | d |
|  | Deltamethrin | 18.80±3.61 | b | 19.8±4.18 | d |
| **An.**  **gambiae s.s.** | Ctrl_Fung | 0 | a | 0 | a |
|  | Ctrl_Pyr | 0 | a | 0 | a |
|  | Met_RFP | 0 | a | 54.9±4.10 | b |
|  | Met_Hyb | 0 | a | 90.6±3.55 | c |
|  | Permethrin | 9.30±2.03 | b | 9.30±2.03 | d |
|  | Deltamethrin | 6.72±2.81 | b | 8.95±3.99 | d |
| **An.**  **kisumu** | Ctrl_Fung | 0 | a | 1.00±1.00 | a |
|  | Ctrl_Pyr | 0 | a | 0 | a |
|  | Met_RFP | 0 | a | 72.5±5.50 | b |
|  | Met_Hyb | 0 | a | 96.4±2.06 | c |
|  | Permethrin | 100 | b | 100 | c |
|  | Deltamethrin | 100 | b | 100 | c |

**S1 Table Legend:** Mortality percentage of mosquitoes over 24 h and a week observation after 1 h exposure in WHO cylinder to insecticides and fungi. SE: Standard error of the mean; ^2^Pairwise comparison of LT_50_ values: fungi without letters in common are significant at P<0.05
