# Supplementary material for: Transgenic Metarhizium pingshaense synergistically ameliorates pyrethroid-resistance in wild-caught, malaria-vector mosquitoes
Source: PLoS One. 2018 Sep 7;13(9):e0203529. doi: 10.1371/journal.pone.0203529 (PMC6128571; doi:10.1371/journal.pone.0203529)
Supplement: S2 Table — (DOCX) [file pone.0203529.s004.docx]

| **Fungal Treatment** | **Species** | **Days Post Infection** | **Pesticide** | **Mean Time Flying (%)** | **Standard error (%)** |
| --- | --- | --- | --- | --- | --- |
| RFP | An.coluzzii | 1 | Permethrin | 28.8 | 4.58 |
| RFP | An.gambiae s.s. | 1 | Permethrin | 23.8 | 2.19 |
| RFP | An.kisumu | 1 | Permethrin | 100.0 | 0.00 |
| RFP | An.coluzzii | 2 | Permethrin | 31.1 | 1.51 |
| RFP | An.gambiae s.s. | 2 | Permethrin | 27.4 | 2.98 |
| RFP | An.kisumu | 2 | Permethrin | 100.0 | 0.00 |
| RFP | An.coluzzii | 3 | Permethrin | 32.2 | 2.14 |
| RFP | An.gambiae s.s. | 3 | Permethrin | 33.3 | 2.88 |
| RFP | An.kisumu | 3 | Permethrin | 100.0 | 0.00 |
| RFP | An.coluzzii | 4 | Permethrin | 32.1 | 3.56 |
| RFP | An.gambiae s.s. | 4 | Permethrin | 33.1 | 1.06 |
| RFP | An.kisumu | 4 | Permethrin | 100 | 0.00 |
| RFP | An.coluzzii | 5 | Permethrin | 44.9 | 3.05 |
| RFP | An.gambiae s.s. | 5 | Permethrin | 50.6 | 3.67 |
| RFP | An.kisumu | 5 | Permethrin | 100.0 | 0.00 |
| Hybrid | An.coluzzii | 1 | Permethrin | 24.4 | 3.27 |
| Hybrid | An.gambiae s.s. | 1 | Permethrin | 23.0 | 1.23 |
| Hybrid | An.kisumu | 1 | Permethrin | 100 | 0.00 |
| Hybrid | An.coluzzii | 2 | Permethrin | 31.1 | 3.52 |
| Hybrid | An.gambiae s.s. | 2 | Permethrin | 32.5 | 2.02 |
| Hybrid | An.kisumu | 2 | Permethrin | 100 | 0.00 |
| Hybrid | An.coluzzii | 3 | Permethrin | 42.1 | 2.62 |
| Hybrid | An.gambiae s.s. | 3 | Permethrin | 40.7 | 3.11 |
| Hybrid | An.kisumu | 3 | Permethrin | 100 | 0.00 |
| Hybrid | An.coluzzii | 4 | Permethrin | 54.8 | 4.73 |
| Hybrid | An.gambiae s.s. | 4 | Permethrin | 54.2 | 3.74 |
| Hybrid | An.kisumu | 4 | Permethrin | 100 | 0.00 |
| Hybrid | An.coluzzii | 5 | Permethrin | 91.5 | 3.59 |
| Hybrid | An.gambiae s.s. | 5 | Permethrin | 86.1 | 8.24 |
| Hybrid | An.kisumu | 5 | Permethrin | 100 | 0.00 |
| Control | An.coluzzii | 1 | Permethrin | 19.7 | 1.64 |
| Control | An.gambiae s.s. | 1 | Permethrin | 26.0 | 2.25 |
| Control | An.kisumu | 1 | Permethrin | 100 | 0.00 |
| Control | An.coluzzii | 2 | Permethrin | 19.2 | 2.52 |
| Control | An.gambiae s.s. | 2 | Permethrin | 25.3 | 2.26 |
| Control | An.kisumu | 2 | Permethrin | 100 | 0.00 |
| Control | An.coluzzii | 3 | Permethrin | 21.9 | 2.33 |
| Control | An.gambiae s.s. | 3 | Permethrin | 23.3 | 2.75 |
| Control | An.kisumu | 3 | Permethrin | 100 | 0.00 |
| Control | An.coluzzii | 4 | Permethrin | 17.7 | 1.96 |
| Control | An.gambiae s.s. | 4 | Permethrin | 22.8 | 1.92 |
| Control | An.kisumu | 4 | Permethrin | 100 | 0.00 |
| Control | An.coluzzii | 5 | Permethrin | 23.2 | 1.81 |
| Control | An.gambiae s.s. | 5 | Permethrin | 23.9 | 2.03 |
| Control | An.kisumu | 5 | Permethrin | 100 | 0.00 |
| RFP | An.coluzzii | 1 | No Pesticide | 0.00 | 0.00 |
| RFP | An.gambiae s.s. | 1 | No Pesticide | 0.00 | 0.00 |
| RFP | An.kisumu | 1 | No Pesticide | 0.00 | 0.00 |
| RFP | An.coluzzii | 2 | No Pesticide | 0.00 | 0.00 |
| RFP | An.gambiae s.s. | 2 | No Pesticide | 0.00 | 0.00 |
| RFP | An.kisumu | 2 | No Pesticide | 0.00 | 0.00 |
| RFP | An.coluzzii | 3 | No Pesticide | 0.00 | 0.00 |
| RFP | An.gambiae s.s. | 3 | No Pesticide | 0.00 | 0.00 |
| RFP | An.kisumu | 3 | No Pesticide | 0.00 | 0.00 |
| RFP | An.coluzzii | 4 | No Pesticide | 0.00 | 0.00 |
| RFP | An.gambiae s.s. | 4 | No Pesticide | 0.00 | 0.00 |
| RFP | An.kisumu | 4 | No Pesticide | 0.00 | 0.00 |
| RFP | An.coluzzii | 5 | No Pesticide | 0.00 | 0.00 |
| RFP | An.gambiae s.s. | 5 | No Pesticide | 0.00 | 0.00 |
| RFP | An.kisumu | 5 | No Pesticide | 0.00 | 0.00 |
| Hybrid | An.coluzzii | 1 | No Pesticide | 0.00 | 0.00 |
| Hybrid | An.gambiae s.s. | 1 | No Pesticide | 0.00 | 0.00 |
| Hybrid | An.kisumu | 1 | No Pesticide | 0.00 | 0.00 |
| Hybrid | An.coluzzii | 2 | No Pesticide | 0.00 | 0.00 |
| Hybrid | An.gambiae s.s. | 2 | No Pesticide | 0.00 | 0.00 |
| Hybrid | An.kisumu | 2 | No Pesticide | 0.00 | 0.00 |
| Hybrid | An.coluzzii | 3 | No Pesticide | 0.00 | 0.00 |
| Hybrid | An.gambiae s.s. | 3 | No Pesticide | 1.79 | 1.04 |
| Hybrid | An.kisumu | 3 | No Pesticide | 2.79 | 1.83 |
| Hybrid | An.coluzzii | 4 | No Pesticide | 5.96 | 1.62 |
| Hybrid | An.gambiae s.s. | 4 | No Pesticide | 15.2 | 1.78 |
| Hybrid | An.kisumu | 4 | No Pesticide | 18.0 | 4.14 |
| Hybrid | An.coluzzii | 5 | No Pesticide | 20.2 | 3.42 |
| Hybrid | An.gambiae s.s. | 5 | No Pesticide | 23.8 | 0.58 |
| Hybrid | An.kisumu | 5 | No Pesticide | 22.4 | 1.01 |
| Control | An.coluzzii | 1 | No Pesticide | 0.00 | 0.00 |
| Control | An.gambiae s.s. | 1 | No Pesticide | 0.00 | 0.00 |
| Control | An.kisumu | 1 | No Pesticide | 0.00 | 0.00 |
| Control | An.coluzzii | 2 | No Pesticide | 0.00 | 0.00 |
| Control | An.gambiae s.s. | 2 | No Pesticide | 0.00 | 0.00 |
| Control | An.kisumu | 2 | No Pesticide | 0.00 | 0.00 |
| Control | An.coluzzii | 3 | No Pesticide | 0.00 | 0.00 |
| Control | An.gambiae s.s. | 3 | No Pesticide | 0.00 | 0.00 |
| Control | An.kisumu | 3 | No Pesticide | 0.00 | 0.00 |
| Control | An.coluzzii | 4 | No Pesticide | 0.00 | 0.00 |
| Control | An.gambiae s.s. | 4 | No Pesticide | 0.00 | 0.00 |
| Control | An.kisumu | 4 | No Pesticide | 0.00 | 0.00 |
| Control | An.coluzzii | 5 | No Pesticide | 0.00 | 0.00 |
| Control | An.gambiae s.s. | 5 | No Pesticide | 0.00 | 0.00 |
| Control | An.kisumu | 5 | No Pesticide | 0.00 | 0.00 |

**S2 Table Legend:** Results of World Health Organization (WHO) susceptibility tests of the impact of fungal infection on insecticide-susceptibility of wild caught mosquitoes from Burkina Faso and laboratory *Anopheles gambiae Kisumu strain*
